# ANDDigest: a new web-based module of ANDSystem for the search of knowledge in the scientific literature

**DOI:** 10.1186/s12859-020-03557-8

**Published:** 2020-09-14

**Authors:** Timofey V. Ivanisenko, Olga V. Saik, Pavel S. Demenkov, Nikita V. Ivanisenko, Alexander N. Savostianov, Vladimir A. Ivanisenko

**Affiliations:** 1 Laboratory of Computer-Assisted Proteomics, Institute of Cytology & Genetics, Siberian Branch, Russian Academy of Sciences, Prospekt Lavrentyeva 10, Novosibirsk, 630090 Russia; 2 Laboratory of Computer Genomics, Novosibirsk State University, st. Pirogova 1, Novosibirsk, 630090 Russia; 3 Kurchatov Genomics Center, Institute of Cytology & Genetics, Siberian Branch, Russian Academy of Sciences, Prospekt Lavrentyeva 10, Novosibirsk, 630090 Russia; 4 Novosibirsk State University, st. Pirogova 1, Novosibirsk, 630090 Russia

**Keywords:** Text-mining, Web-based tool, Knowledge retrieval, Information search, Associative gene network, Dynamics of interest, Trend analysis

## Abstract

**Background:**

The rapid growth of scientific literature has rendered the task of finding relevant information one of the critical problems in almost any research. Search engines, like Google Scholar, Web of Knowledge, PubMed, Scopus, and others, are highly effective in document search; however, they do not allow knowledge extraction. In contrast to the search engines, text-mining systems provide extraction of knowledge with representations in the form of semantic networks. Of particular interest are tools performing a full cycle of knowledge management and engineering, including automated retrieval, integration, and representation of knowledge in the form of semantic networks, their visualization, and analysis. STRING, Pathway Studio, MetaCore, and others are well-known examples of such products. Previously, we developed the Associative Network Discovery System (ANDSystem), which also implements such a cycle. However, the drawback of these systems is dependence on the employed ontologies describing the subject area, which limits their functionality in searching information based on user-specified queries.

**Results:**

The ANDDigest system is a new web-based module of the ANDSystem tool, permitting searching within PubMed by using dictionaries from the ANDSystem tool and sets of user-defined keywords. ANDDigest allows performing the search based on complex queries simultaneously, taking into account many types of objects from the ANDSystem’s ontology. The system has a user-friendly interface, providing sorting, visualization, and filtering of the found information, including mapping of mentioned objects in text, linking to external databases, sorting of data by publication date, citations number, journal H-indices, etc. The system provides data on trends for identified entities based on dynamics of interest according to the frequency of their mentions in PubMed by years.

**Conclusions:**

The main feature of ANDDigest is its functionality, serving as a specialized search for information about multiple associative relationships of objects from the ANDSystem’s ontology vocabularies, taking into account user-specified keywords. The tool can be applied to the interpretation of experimental genetics data, the search for associations between molecular genetics objects, and the preparation of scientific and analytical reviews. It is presently available at https://anddigest.sysbio.ru/.

## Introduction

The vast amounts of published biomedical literature has made extremely urgent the problem of an effective automated finding of necessary information. The popular search engines, such as Google Scholar, PubMed, Scopus, and Web of Knowledge, are powerful universal tools for keyword-based document search without linking to any specific field of knowledge [1–3]. Text-mining of scientific publications is an alternative to such searching, providing automated extraction and formalized representation of specific information [4].

Text-mining-based systems are commonly dedicated to the solving of two types of tasks: the tasks of named-entity recognition (NER) and relationship extraction (RE). NER tasks consist of recognizing the names of all entities mentioned in text. Such objects are usually linked to the corresponding entries in external databases, featuring descriptions of their structural and functional properties. For example, proteins/genes are often associated with Uniprot/Entrez gene databases [5, 6] and chemical compounds with PubChem [7]. The RE task is comprised of identification of all relationships between the recognized objects mentioned in a document.

Many methods have been proposed for dealing with a NER problem, based both on the use of manually specified rules and templates, and on traditional machine-learning approaches. For example, the POSBioTM-NER system [8] provides an automated recognition of biomedical terms by combining support vector machine (SVM) and conditional random field (CRF) approaches, while the information surrounding morphological features, parts of speech, and collocations of words are used as a set of features. Finkel and colleagues [9] used the features of the analysed texts and external resources to determine the biomedical named objects. ABNER [10] and Gimli [11] are examples of open-source CRF-based NER tools. With this, Tsuruoka et al. [12] developed a system for mapping of biomedical sequences, entitled GENIA Tagger, by employing the maximum entropy model and tagging algorithm. In the work of Chang et al. [13], the authors introduced biomedical word embeddings into the CRF model as additional input data, which allowed them to achieve significant improvement of the model. PowerBioNE is a hidden Markov model (HMM)-based NER system developed by Zhou et al. [14], which used various evidential features to deal with a naming conventions problem, while the K-nearest neighbour (KNN) approach was applied for solving of the data sparseness problem. NERBio [15] is an example of a rule-based system. The skip-chain CRF tool, proposed by Liao and Wu [16], implements the conventional machine-learning method, which is a representative of traditional machine-learning approaches. Wei et al. [17] proposed the combined use of a bidirectional long short-term memory network (BiLSTM) and CRF, which showed better results in comparison with rule-based and conventional-based systems. This approach provides a more complete contextual information and allows for more effective dealing with problems related to the inability to handle the strong dependence on tags in the sequence. Deep neural networks also are used in NER tasks. In particular, with the HUNER system [18], the LSTM-CRF approach was employed for dealing with these tasks.

Various methods are also being used to solve the RE problem, such as, for example, co-occurrence, template-based or rule-based approaches, along with machine learning, including feature-based, kernel-based, recurrent neural networks (RNN), and others. At the basis of co-occurrence-based approaches lies the assumption that keywords co-occurring together in the same text can be functionally related [19]. Such methods allow performing just an analysis of frequencies of occurrence of keywords in text. At the same time, they do not provide any information regarding the type of relationship between the concepts corresponding to keywords. Also, such methods normally have lower precision in comparison to natural language processing (NLP)-based algorithms [20]. However, despite the aforementioned shortcomings, they have earned great practical use because of simplicity [21].

The first generations of template-based systems utilized regular expressions to match words describing relationships between entities [22]. Most of such templates consisted of names of objects and additional sets of keywords representing relationships between them, for example “trigger” and “stimulate.” Later generations of tools additionally began to use other approaches, such as part-of-speech (POS) tagging [23]. At the same time, many rule-based systems also applied various constraints for dealing with challenging issues in terms of expression by templates, such as negative relationships [24]. Some rule-based systems distance themselves from template-based approaches by replacing regular expressions with heuristic algorithms and sets of procedures [25]. Unlike the co-occurrence-based methods, the use of manually defined rules and templates often allows authors to achieve high values of accuracy, but they tend to have low completeness [26]. Several automated methods of rules and templates generation were proposed for dealing with this problem [27, 28]. RLIMS-P [29, 30] and MinePhos [31] are examples of template-based tools, both using rule-based templates for mining information on phosphorylation from the literature.

Most of the machine-learning (ML) methods, commonly employed in RE tasks, typically require a large set of annotated biomedical cases (supervised learning). These textual corpora usually pass pre-processing by NLP tools and then are applied as training sets for the construction of classification models. Among the Machine Learning methods, the most widely known are Naive Bayesian Classifier, HMM, SVM, Artificial Neural Networks (ANN), etc. [32–34]. These classifiers can use various functions intended to represent different characteristics of data (e.g., shortest path, bag-of-words (BOW), POS tagging) [35].

In recent years, deep-learning techniques, such as RNN, have proved to lead to outstanding results for various NLP tasks, including the RE. Considerable increases in performance were produced using the convolutional [36, 37] and recurrent neural networks [38] approaches. The success of deep learning in biomedical NLP is partly related to the development of vector models of words, such as Word2Vec [39] and, more recently, ELMo [40], BERT [41], GPT [42], Transformer-XL [43], and GPT-2 [44]. These models learn word vector representations, capturing the syntactic and semantic relationships of words, and are known as word embeddings.

Among the computer programs that apply text-mining methods for the automated extraction of biomedical knowledge, special attention should be paid to information systems implementing a so-called full cycle of engineering and knowledge management. This includes retrieval, integration, and presentation of knowledge to the end-user, as well as allowing the performing of various types of additional analysis. The well-known examples of such systems are PathWay Studio [45], MetaCore [46, 47], STRING [48], and others. These tools consist of knowledge bases where the information, obtained from automated analysis of PubMed abstracts, full-text articles, along with various factographic databases, is accumulated. In addition, such systems have graphical user interfaces (GUIs) permitting access to the data stored in knowledge bases via the user-specified queries and performing the reconstruction and analysis of semantic networks on this basis.

The ANDSystem tool [49–52], previously developed by us, is another instance of systems that carry out a full cycle of engineering and management of knowledge. The ANDSystem tool is based on unique, specialized ontology [50], which, in many respects, outperforms the existing analogues by the description of a subject area.

In particular, using the ANDSystem tool, a number of studies were performed, including an analysis of data from high-performance proteomic experiments on the study of Helicobacter pylori and their relationship with the development of gastritis and tumours of the stomach [53]; an analysis of the proteomic urine profile of a healthy person under normal conditions and under the influence of space flight factors [54, 55]; analysis of the tissue-specific effects of gene knockout and the search for potential targets for drugs [56]; analysis of the molecular mechanisms of comorbidity of disease [57–59]; the search for novel tuberculosis susceptibility candidate genes [60]; analysis of the interactions of hepatitis C virus proteins with human proteins [61]; reconstruction and analysis of mosaic gene networks [62], etc. Moreover, with the prioritization methods of the ANDSystem tool, the key genes involved in the formation of a comorbid state of asthma and hypertension were identified [59], followed by experimental confirmation of their role in pathology [63]. Based on ANDSystem’s knowledge base, we developed the web-based FunGeneNet tool. It facilitates estimating enrichment of functional interactions in experimental gene sets [64]. The NACE program also uses the ANDSystem’s base of knowledge for the assessment of the effectiveness of potential signal transduction in gene networks [65].

However, like similar tools, the knowledge base of the ANDSystem is composed by a preliminary analysis of the literature within the used ontology. The search for information with a combined approach, which leverages methods of knowledge extraction in the frames of the specified ontology in combination with a list of user-defined keywords, can expand the amounts of extracted information or clarify the search.

Textpresso [66] and Polysearch [67] are examples of such systems. Textpresso Central offers a variety of options for the literature search, starting from a simple keyword-based search up to the performance of complex searches based on a wide range of categories or concepts using the dictionaries of names of objects as well as their synonyms within these categories. Furthermore, Textpresso Central utilizes several types of search filters, which allow the user to limit the main pool of the search through the literature. The system also permits sorting the obtained results by date. At the time of writing, PolySearch searches for interactions between many types of objects, including diseases, genes/proteins, drugs, metabolites, SNPs, pathways, and tissues. The search employs multiple information sources, including PubMed, OMIM [68], DrugBank [69], and Swiss-Prot [70]. Among the other systems that provide similar functionality are XplorMed [71], MedlineR [72], LitMiner [73].

This paper describes a new web-based module of the ANDSystem tool, entitled ANDDigest, which enables performing searches of information in PubMed using dictionaries of the ANDSystem tool as well as user-defined keywords. ANDDigest facilitates conducting searches based on complex queries that simultaneously take into account the conditions for multiple interactions between different types of objects from the ANDSystem ontology. The GUI of ANDDigest supplied the found information both in text form with mapped objects as well as in the form of associative networks. The interface features a wide range of tools, providing various types of filtering of found scientific articles (by publication date, journal’s H-index, etc.). Moreover, ANDDigest permits computing the dynamics of the level of interest to the specific object in the scientific literature, as well as sorting of found documents by the average score of interest according to user-defined entities.

### Implementation

The text-mining module of the ANDSystem tool ensures all the necessary pre-processing of the textual data, including the conversion of text to the ANDSystem format, dividing it into separated sentences, normalization, morphological, and syntactic analysis, and mapping of the named entities (object names). In terms of the named entity recognition, a complex dictionary-based algorithm was implemented in the ANDSystem tool. All the prepared texts are then transferred for automated extraction of information about interactions between the found objects using the semantic-linguistic templates. In total, the ANDSystem tool contains more than 10,000 of such manually created templates, allowing the establishment of more than 25 form of relationships between 13 types of objects (proteins, genes, metabolites, microRNAs, biological processes, phenotypic traits, drugs, and their side effects, diseases, and others). The ANDSystem tool considers such interactions as physical interactions with the formation of molecular complexes (protein/protein, protein/DNA, metabolite/protein); catalytic reactions and proteolytic events involving the substrate/enzyme/product, as along with transformations of the substrate/product in the case of complex reactions with a lack of information surrounding the involved enzymes; gene co-expression; side effects of drugs; associative relationships of genes, proteins, metabolites, biological processes, phenotypic traits with diseases, etc. Regulatory interactions are divided into positive and negative regulation, including regulation of gene expression, regulation of protein translation, regulation of protein function/activity, regulation of stability and degradation of proteins, and regulation of transporting of substances. Such regulation can be carried out with the participation of proteins, metabolites, drugs, and miRNAs. Each interaction is characterized by the involved participants, their types, direction, and by cells and organisms along with where the event was described in the literature. The knowledge base of the ANDSystem tool was created based on a large-scale automatic analysis of more than 25 million texts of PubMed abstracts and dozens of factographic databases in the field of biology and biomedicine. It is a unique resource containing formalized information about more than 20 million interactions. It is worth noting that unlike other similar systems, in the ANDSystem tool, proteins and genes are considered separate entities, interconnected by a directed link (gene- > protein)-expression. All genes in the ANDSystem tool have a special attribute that characterizes tissue-specific expression, which permits the reconstruction of gene networks, including only those genes that are expressed in a given tissue, i.e., gene networks that are functioning in a given tissue.

### Extraction and scoring of co-occurrence associations

The new version of the ANDSystem tool, extended by the ANDDigest module, allows performing a co-occurrence-based information search considering user-specified keywords. Such a type of search was not used in the ANDSystem tool previously. We score associations between objects from the ANDSystem ontology using the co-occurrence-based text-mining scoring scheme, implemented in STRING [74] and widely used by a number of other systems [75–77]. In our case, we consider the co-occurrence values between all pairs of objects from the ontology of the ANDSystem tool (*O*(*i*_1,_*j*_1_), *O*(*i*_2,_*j*_2_)), where *i*_1_ = (1, *NT*), *j*_1_ = (1, *N*(*i*_1_)), *i*_2_ = (1, *NT*), *j*_2_ = (1, *N*(*i*_2_)), *NT* – is a number of all types of objects from the ontology of the ANDSystem tool, *N*(*i*) – the number of all objects of *i*_*th*_ type, stored in the ontology of the ANDSystem tool, and *j*_1_ ≠ *j*_2_ when *i*_1_ = *i*_2_. For each pair of objects, a weighted indicator of their co-occurrence was calculated based on the level of abstracts as well as individual sentences over the n abstracts in the text corpus:

1 $$ C\left(O\left({i}_{1,}{j}_1\right),O\left({i}_{2,}{j}_2\right)\right)=\sum \limits_{k=1}^n\left[{\upomega}_s{\updelta}_{sk}\left(O\left({i}_{1,}{j}_1\right),O\left({i}_{2,}{j}_2\right)\right)+{\upomega}_a{\updelta}_{ak}\left(O\left({i}_{1,}{j}_1\right),O\left({i}_{2,}{j}_2\right)\right)\right], $$

where ω_*a*_ =3 and ω_*s*_ =0.2 are co-occurrence weights within the same abstract and same sentence, respectively, and δ_*sk*_ and δ_*ak*_ are equal to 1 or 0 depending on whether or not *O*(*i*_1,_*j*_1_) and *O*(*i*_2,_*j*_2_) objects co-occur in abstract k or a sentence within it.

A co-occurrence score (s-score) was calculated as:

2 $$ S\left(O\left({i}_{1,}{j}_1\right),O\left({i}_{2,}{j}_2\right)\right)=C{\left(O\left({i}_{1,}{j}_1\right),O\left({i}_{2,}{j}_2\right)\right)}^{\upalpha}{\left(\frac{C\left(O\left({i}_{1,}{j}_1\right),O\left({i}_{2,}{j}_2\right)\right)C\left(O\left({i}_1\right),O\left({i}_2\right)\right)}{C\left(O\left({i}_{1,}{j}_1\right),O\left({i}_2\right)\right)C\left(O\left({i}_1\right),O\left({i}_{2,}{j}_2\right)\right)}\right)}^{1-\upalpha}, $$

where *C*(*O*(*i*_1,_*j*_1_), *O*(*i*_2_)) is a sum over all *O*(*i*_2_) objects of the *i*_2_-th type paired with an *O*(*i*_1,_*j*_1_) object, *C*(*O*(*i*_1_), *O*(*i*_2,_*j*_2_)) is the sum over all *O*(*i*_1_) objects of the *i*_1_-th type paired with an *O*(*i*_2,_*j*_2_) object, the normalizing factor, *C*(*O*(*i*_1_), *O*(*i*_2_)), is the sum over all pairs of objects of the *i*_1_-th and *i*_2_-th type, and the weighting factor α = 0.6. The values for ω_*s*_, ω_*a*_, and α parameters were obtained from [74].

### Assessment of accuracy

To assess the quality of the text-mining results, we constructed two reference sets, based on the gene-disease and protein-protein interactions. A positive sample, containing gene-disease associations, was created using a curated part of the DisGeNET database (version 6.0) [78] and included over 33,000 pairs. A negative sample contained over 2,000,000 pairs. It was formed from random genes and diseases from the ANDSystem’s ontology, excluding those interactions that were presented in the positive sample or the database of the ANDSystem tool.

**Fig. 1:**
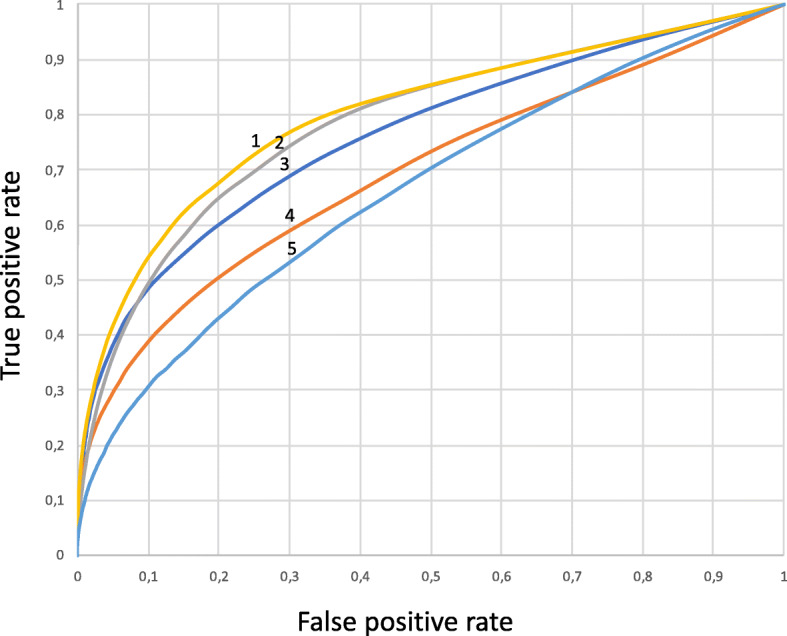
The ROC curve illustrating the accuracy of obtained text-mining results for different types of interaction. (1) drug-drug interactions (AUC = 87.4%), (2) disease-disease interactions (AUC = 85.2%), (3) protein-protein interactions (AUC = 79.6%), (4) gene-disease interactions (AUC = 73.9%), (5) cells-drug interactions (AUC = 68.4%)

It should be noted that the formed negative sample could contain real pairs of interacting genes and diseases that were not recognized by our templates and were not presented in the curated part of the DisGeNET database. Despite the fact that this circumstance could have a negative impact on the assessment results, we believe that this approach can be considered applicable for our purposes in case if acceptable accuracy indicators will be achieved. When analyzing these samples, the AUC appeared to be 73.9% (Fig. 1).

It should be noted that AUC calculations using a negative sample, containing an equal number of pairs as in a positive sample, showed that the AUC values ​​remained almost unchanged, varying within only 0.1%.

To extract information, the ANDSystem tool uses semantic-linguistic templates with taking into account the grammatical structure of the sentence and the presence of keywords [50, 51]. At the same time these templates do not rely on the frequency of occurrence of protein-protein pairs in the text. In this regard, we suggested that the information on interactions between objects, extracted using the ANDSystem’s templates, can be used as a positive sample for the evaluation of the used co-occurrence-based approach.

The AUC value calculated using a positive sample (262,084 pairs) generated based on gene-disease interactions taken from the ANDSystem tool turned out to be 70.5%. The accuracy based on these data turned out to be even slightly lower than when using the curated gene-disease associations. This may be since gene-disease pairs rarely mentioned in the literature appeared to be among the positive interactions. Thus, such an approach to the formation of a positive sample can be used for a rough estimation of the method based on compatibility. The AUC value calculated using a positive sample based on the gene-disease interactions, extracted only from the ANDSystem database (262,084 gene-disease pairs), appeared to be 70.5%. I.e., obtained accuracy appeared to be even lower than compared to the gene-disease associations formed on the basis of the curated data. This may be probably related to the fact that gene-disease pairs rarely mentioned in the literature could be among the positive interactions. Thus, such an approach to the formation of a positive sample can be used for a rough estimation of our co-occurrence-based method.

The testing sample for protein-protein interactions was formed in the opposite way compared to the gene-disease described above. The negative sample contained pairs of non-interacting human proteins, obtained from the [79]. Only those proteins that were found together in at least one PubMed abstract [80] were considered. Thus, 14,430 pairs of proteins were included in the negative sample. A positive sample was formed according to the protein-protein interactions presented in the ANDSystem knowledge base. Only such pairs of proteins were considered, the interactions between which were represented in the ANDSystem tool by the “interaction” type, characterized by physical interactions between proteins with the formation of complexes. In total, the positive sample contained 179,215 pairs of interactions of human proteins. The AUC value was 79.6%; an accuracy estimation on samples with the equal number of protein pairs in the negative and positive samples showed approximately the same AUC value, equal to 79.6% (Fig. 1).

We estimated the co-occurrence method for all types of the ANDSystem tool interactions by generating the positive samples based on the information from the ANDSystem’s templates, and negative samples, formed from random pairs of objects that were found together in the text at least once. A total of 47 pairwise interaction combinations were analyzed for 12 types of objects. AUC values ranged from 68.4% (cells-drug interactions) to 87.4% (drug-drug interactions). In Fig. 1 the ROC curves for interactions having different AUC values are shown. The values for the mean and standard deviations of negative sets were used to calculate the z-score and estimate the *p*-value for each interaction.

### Calculation of trend strength

For each object from the ANDSystem ontology, an indicator of interest, based on the dynamics of its mentions in PubMed publications by years, was calculated. The dynamics were calculated in two ways: in the first case, only the absolute numbers of articles containing the object by each year were considered. In the second case, the number of publications concerning the object in each year was normalized to the total number of articles published in PubMed during that year. The trend strength estimations were performed based on the normalized dynamics using the non-parametric Mann-Kendall test [81, 82]. The analysis was performed with the *mk.test* function from the *trend v1.1.1* package [83] of the R programming language and provided the values of trend strength (S) and statistical significance (z-score and *p*-value).

## Results

### The user interface of ANDDigest

The web-based ANDDigest tool is designed for performing flexible searches of information in the mapped PubMed data based on the ANDSystem tool dictionaries and considering user-defined keywords. Its GUI enables performing searches, visualization, and various forms of sorting and filtering of the results, as well as saving them in different formats.

### The search of information with taking into account the user-defined keywords

**Fig. 2:**
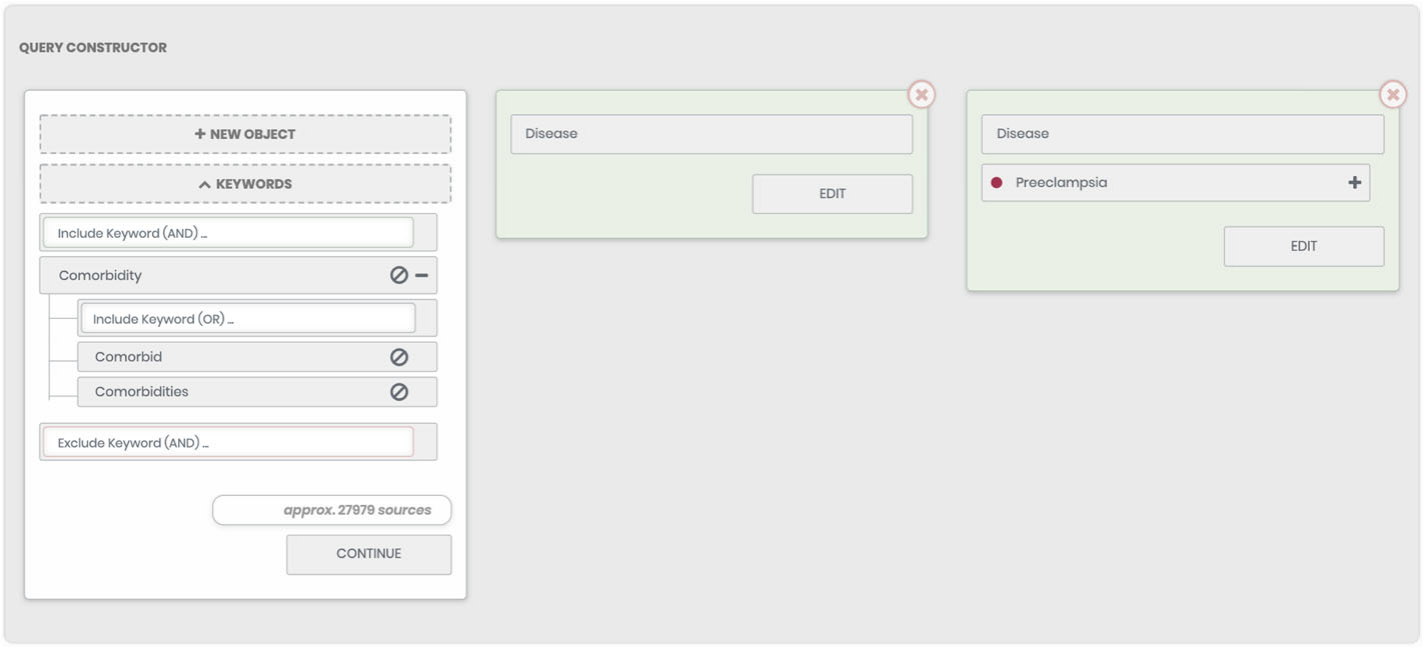
The main window of ANDDigest for the preparation of search queries

The search is performed using the pre-processed text corpus from PubMed [80] containing mapped objects from the ANDSystem ontology. A search query is defined as a list of concepts (types of objects), specific objects of a given type, keywords, which are related to the concept (located in the same sentence as the objects of the concept), as well as free-keywords that can be arbitrarily determined in the text (Fig. 2). At the same time, the query must contain at least one specified object of the chosen type. All objects included in the query can be connected via the logical AND (the “Include the Object” button) or NOT (the “Exclude the Object” button). In addition to objects and concepts, as was mentioned, the user can specify groups of free keywords. The keywords within the same group are interconnected with a logical OR and the logical AND applies to the first word of each group. It is also possible to set a group of keywords for abstracts that should be excluded from the search results. Such groups are considered according to the logical NOT rule with respect to other objects listed in the request. The search result is a list of PubMed abstracts satisfying the user-constructed query. Moreover, the window provides information regarding the approximate number of sources matching the user request. It should be noted that the current version of ANDDigest allows displaying no more than 5000 abstracts when a query is provided without specification of any keywords, and not more than 30,000 abstracts when keywords are specified.

### Visualization of search results

ANDDigest features two options for displaying the search results - the textual and graphical, which includes a hierarchical tree structure and associative network.

**Fig. 3:**
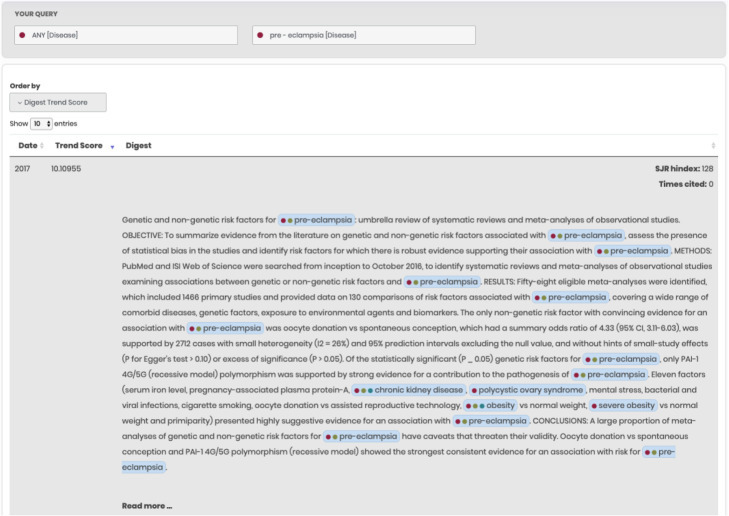
An example of the ANDDigest textual interface

The first type of representation is a table form (Fig. 3). Each line of the table corresponds to a short textual description of the abstract (digest) that satisfies the user’s request and contains mapped objects from the ANDSystem ontology. Clicking on the “read more ...” link displays the full text of the abstract. The table allows sorting digests by various criteria, such as publication date, H-index of the journal, citations number, as well as interest score. Information regarding the H-index was obtained from the SCImago portal [84]. For all journals not presented in SCImago, the Scientific Journal Rankings (SJR) H-index value was set to zero. Information about the publication date and citations number was extracted from the XML version of the PubMed database [80]. The score of interest for the digest (Digest Score) was estimated as the average z-value of all trends of mapped objects in the digest satisfying the user’s query.

**Fig. 4:**
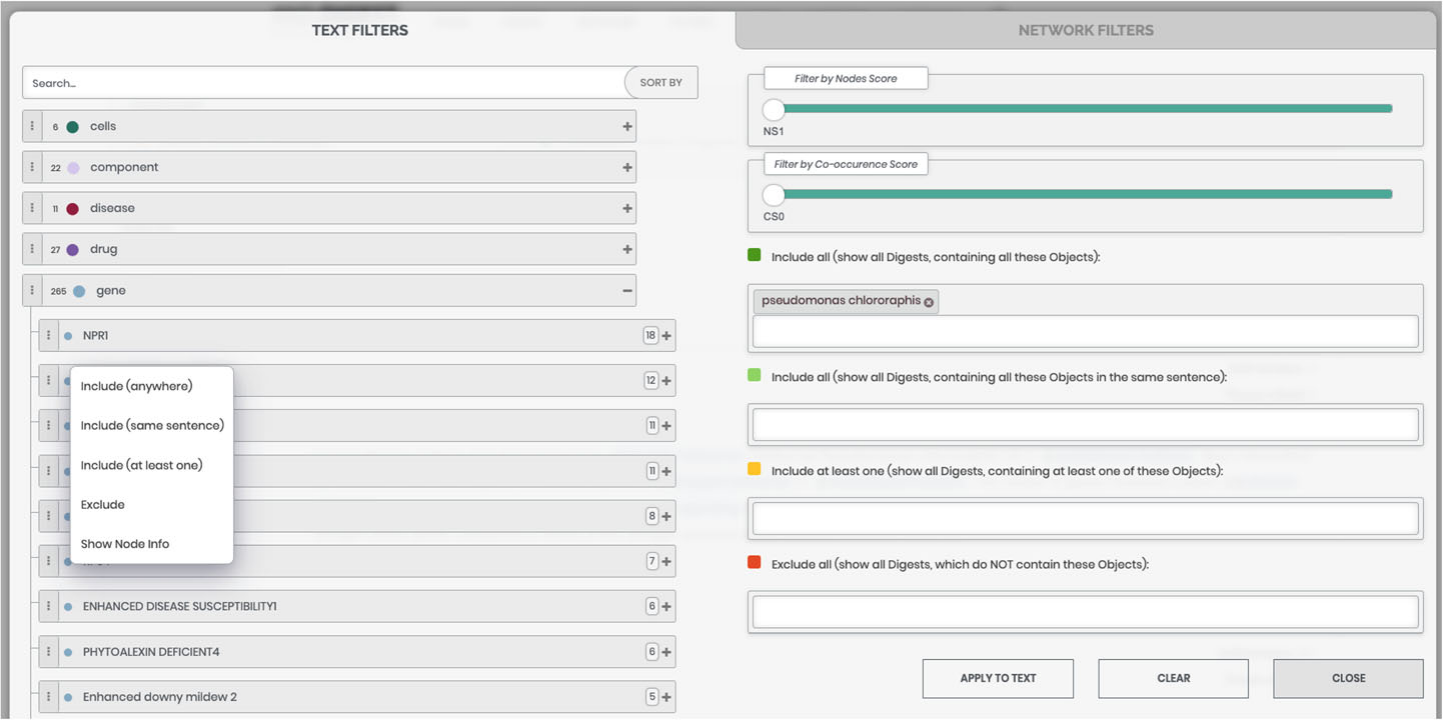
An example of a representation of information in the form of a hierarchical tree in ANDDigest

The representation in the form of a hierarchical tree (Fig. 4) provides the user with information about the mapped objects, grouped by their types, and lists of synonyms for each object recognized in the text. It also permits conducting data sorting and filtering. The sorting can be accomplished by the number of abstracts concerning each object as well as by the trend strength values of the object. In turn, the following options are implemented for filtering: 1) filtering according to the quality of recognition of the object in the text (the values are based on data from the ANDSystem tool knowledge base); and 2) filtering according to the score of pairwise co-occurrences of mapped objects in the text corresponding to the user’s request. Another type of filtering, implemented in the tree form of representations, is a filter based on specifying queries. This filter allows forming subsets of all found digests, based on objects, or their synonyms, selected from the tree as well as manually entered keywords. The selection of objects or synonyms can be made from the context menu located on the left side of the corresponding tree branch (Fig. 4), while keywords can be directly typed into the corresponding text fields. The relationships between the provided entities are automatically determined with logicals AND, OR, and NOT.

Threshold values for filtering by the recognition quality of object names and their co-occurrence can be defined using the appropriate sliders. The range for the first slider is from 1 to 5, where “1” corresponds to the lowest recognition accuracy and “5” the highest. The threshold values for the second slider correspond to the s-score of object co-occurrences. These values range from the minimal to the maximal values among all found pairs of objects, which satisfy the initial user’s search.

**Fig. 5:**
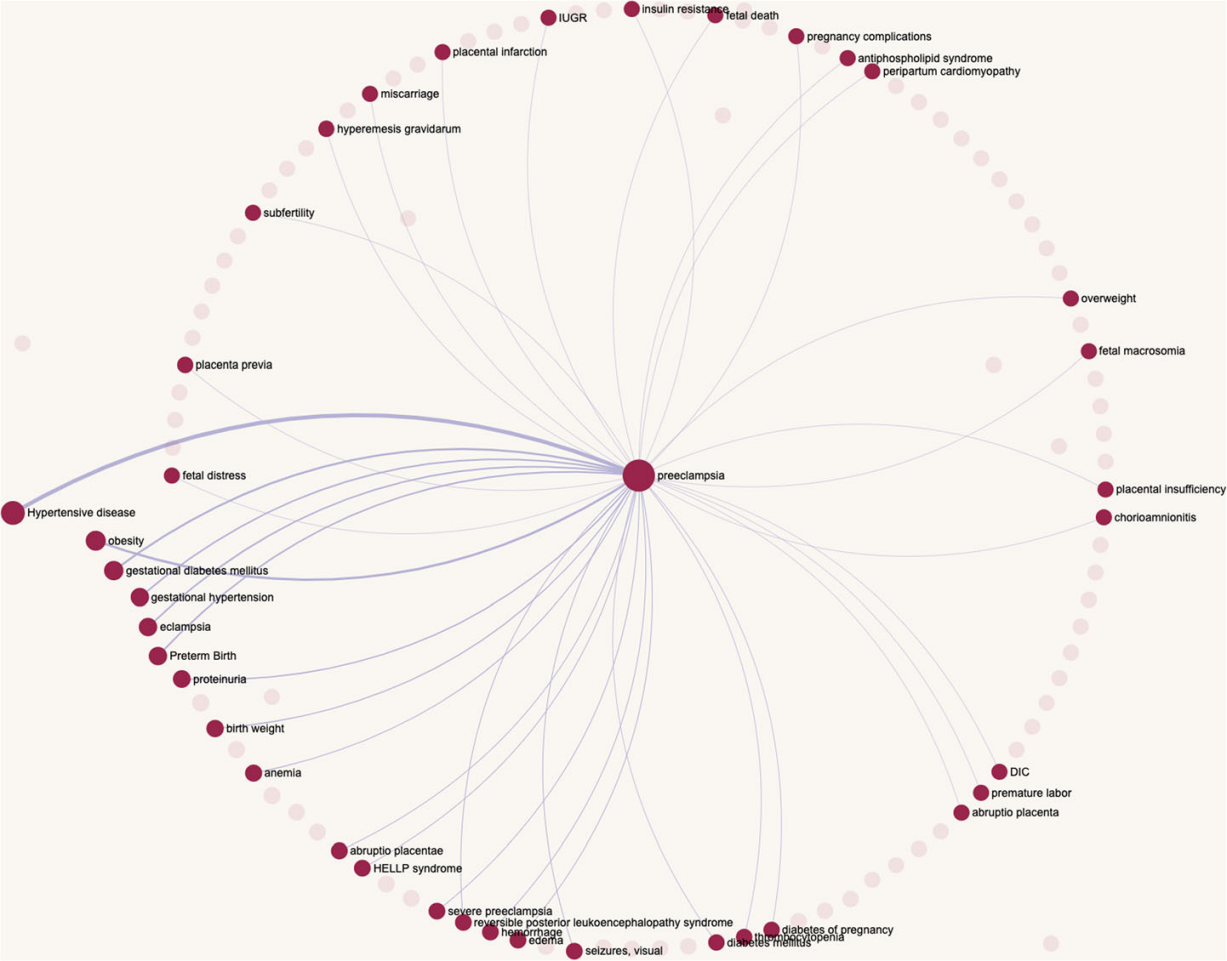
A graphical representation of the list of found diseases associated with preeclampsia in the ANDDigest system. The thickness of the lines reflects the frequency of mentioning of pairs of diseases, and the size of the balls corresponds to the frequency of mentioning of a single disease in the found abstracts. Transparent balls correspond to diseases that were filtered according to the set threshold of compatibility

A graphical representation of the found objects in the form of a network (Fig. 5) is implemented using the sigmajs library [85]. This form illustrates such properties of objects as frequencies of their pairwise (edges) and separated (nodes) occurrences in texts. The filtering of a network by the edge and node sizes can be completed using the appropriate sliders. Right-clicking on the node brings up a context menu. This menu allows performing different layouts of the network, provides information about trend strength of the object, as well as its dynamics of interest, synonyms, and entries in external databases. Besides this, the context menu allows using visible objects on the network as a filter for digests. In this case, relations between visible nodes are set automatically through a logical OR.

### Saving of results

ANDDigest allows the user to download the obtained data considering the applied filters in the JSON, CSV and PDF formats; the network can also be saved as an image in the SVG vector format.

Saving the information in the JSON format provides the user with the opportunity to perform further analysis of the obtained results using various third-party software tools. The JSON file contains a description of two objects: a digests table, entitled anddigest, which includes an id number of the relevant PubMed abstract, the date of publication, the score of interest for the digest, the number of citations, and the mapped text of the digest. The second JSON object is a network, consisting of two parts: nodes and edges. Each node is represented by its internal ANDDigest id, label, list of synonyms, type, colour, size, trend strength value, number of node’s mentions in the text, as well as the node’s coordinates. In turn, the network’s edges are represented by the internal id number, internal identifiers of the first (source) and second (target) nodes, the colour, and the size of the relationship. Further, each edge is presented by the s-score, z-score and *p*-value values reflecting the significance of the co-occurrence of the source and target nodes, as well as with an additional indicator that relationship was also established with the semantic templates of the ANDSystem tool.

The CSV format permits the user to perform a tabular analysis of the obtained data. Like in the case of the JSON format, the file contains mapped results of the query, as well as individual records about the nodes and edges of the network. At the same time, saving in this format does not provide the user with information about the colours of nodes or vertices, their sizes, or their spatial coordinates.

### Examples of the application of the system

The use of the ANDDigest system was demonstrated by analysing diseases comorbid with preeclampsia, as well as by evaluating genes associated with the disease-resistance process with an example of three plants (*Solanum tuberosum*, *Zea ramose,* and *Arabidopsis thaliana*).

For the first example, the entered query was the following: find all articles that mention preeclampsia and any other disease and also contains one of the provided keywords (*Comorbidity* OR *Comorbid* OR *Comorbidities*) (Fig. 2). In the absence of a filtering threshold based on a co-occurrence s-score, the program found 186 diseases associated with preeclampsia mentioned in 57 abstracts.

**Fig. 6:**
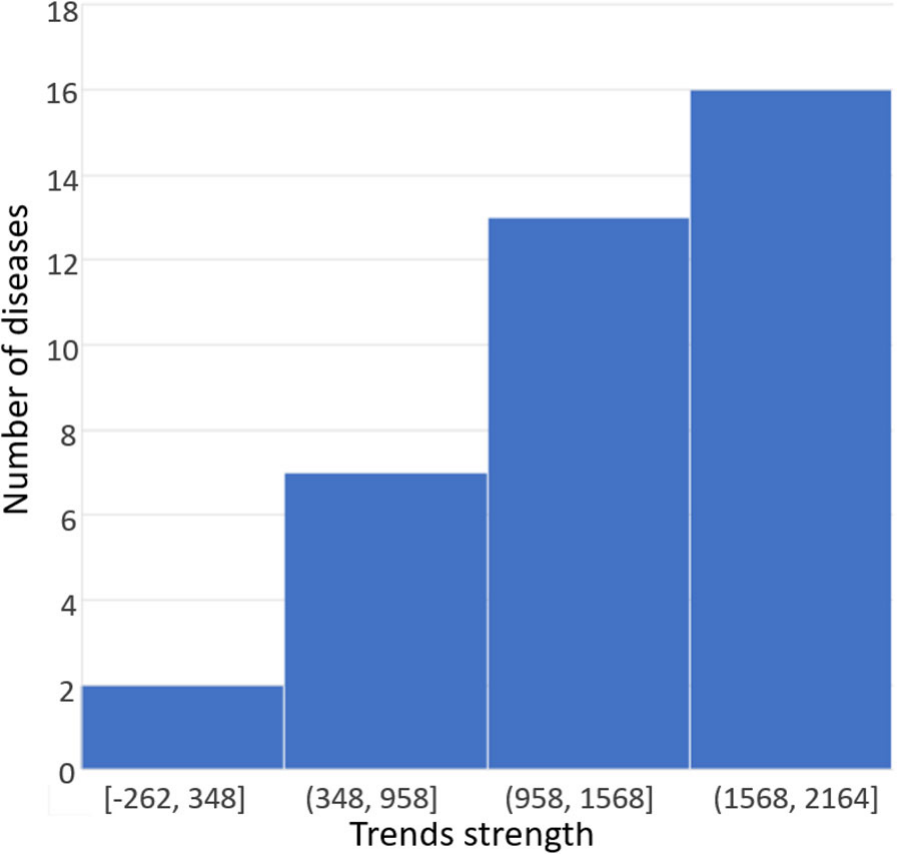
The distribution of the number of diseases associated with preeclampsia by the strength of their trends

After filtering, with a co-occurrence threshold value of s-score = 48 (z-score = 1.65, *p*-value < 0.05), the number of diseases decreased to 38, while the number of abstracts with their mention became 50. The distribution of the number of diseases after the filtration, according to the strength of their trends, is portrayed in Fig. 6.

**Fig. 7:**
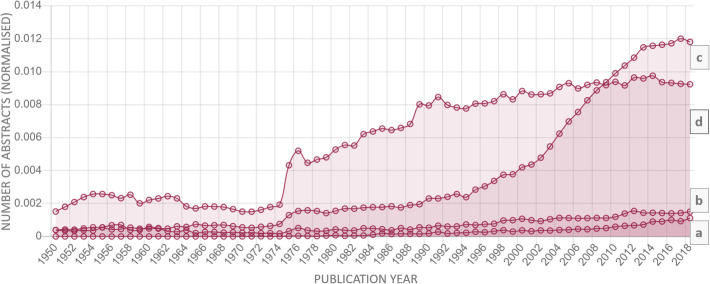
Normalized dynamics of the growth of researchers’ interest in “gestational diabetes” (**a**), “preeclampsia” (**b)**, “obesity” (**c**), and “hypertensive disease” (**d**) by years

Among the remaining diseases, the highest strength value of the trend had “obesity” (2164), while “gestational diabetis” (2059) was in second place and “premature labour” had the lowest value (− 262). It is noteworthy that the trend strength for “preeclampsia” was 1460, which corresponds to the 18th position in the list. Figure 7 depicts the dynamics of interest for “gestational diabetes,” “preeclampsia,” “obesity,” and “hypertensive disease,” in a normalized and non-normalized form.

According to obtained data, it was interesting to observe what genes are mentioned in the literature in the context of two diseases, preeclampsia and obesity, which are known to be comorbid [86]. For this, the following query was used: find all articles containing preeclampsia and obesity, and at least one gene. After performing this query, 144 genes were determined (Filter by Nodes Score = 5) that could potentially be involved in the comorbid state of these two diseases.

At the same time, according to the frequency of mentions of these genes in found abstracts, the “placental growth factor” gene was in the first place (five documents). In second place were “hypoxia-inducible factor 1-alpha” and “C reactive protein,” each of which were linked with three documents.

Placental growth factor (PGF) plays an important role in angiogenesis and vasculogenesis, as well as in embryogenesis [87]. Hypoxia-inducible factor-1 alpha belongs to transcription factors involved in the process of changes in available oxygen in the cellular environment, as well as in angiogenesis, metal transport, mitochondrial function, and cell growth [88, 89]. In turn, C reactive protein interacts with DNA and histones and bestows host defence-associated functions [90].

**Fig. 8:**
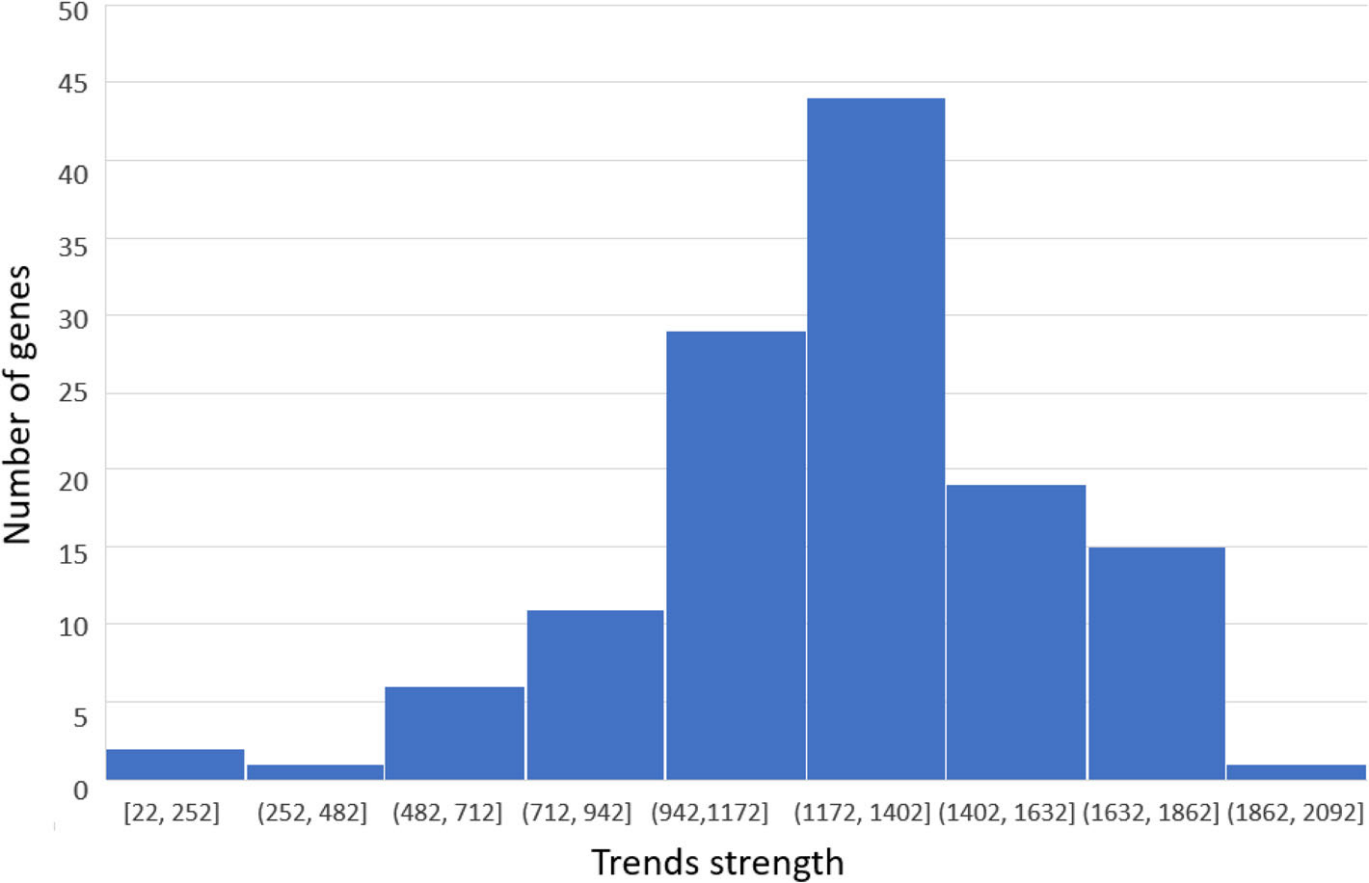
Distribution of the number of genes mentioned together with pre-eclampsia and obesity in texts according to their trend strength

**Fig. 9:**
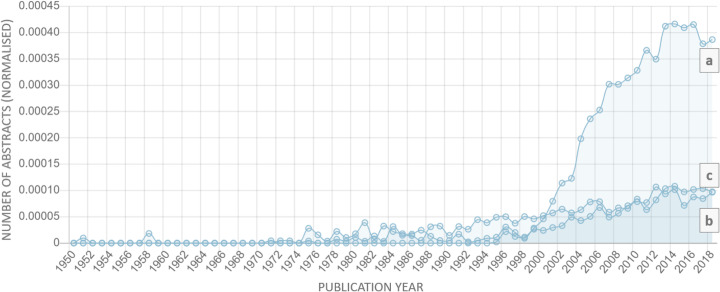
Normalized growth dynamics of researchers’ interest in hypoxia-inducible factor 1-alpha (**a**), PGF (**b**), and C reactive protein (**c**) genes by years

Among the described genes, “C reactive protein” had the greatest trend strength (1796, *p*-value = 1.96E-21), while the hypoxia-inducible factor 1-alpha had the lowest (1324, p-value = 6.6E-16). In turn, the value for PGF was 1681 (p-value = 7.06E-19). It is worthwhile noting that the strength of the trend for all three genes was statistically significant. The grouping of all the found genes by the distribution of trend strengths is shown in Fig. 8, while the dynamics of interest are presented in Fig. 9.

**Fig. 10:**
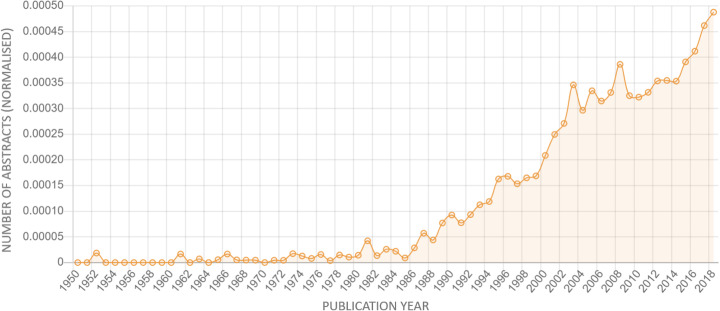
Normalized dynamics of researchers’ interest in the disease-resistance process (trend strength indicator = 1944, *p*-value = 5.51E-24); the lines indicate the linear trends corresponding to the distributions

Another example of the application of ANDDigest was the identification of plant genes associated with the biological process of disease resistance (GO: 0009614). Researchers often face the problem of a lack of information about the functions of genes of the analysed organism and their relationships with biological processes or phenotypic traits. This problem is highly relevant to cultivated plants, such as, for example, *Solanum tuberosum* and Zea ramose. Model plants are of special interest because of an abundant amount of information on genes and their functions provided in the literature. *Arabidopsis thaliana* is a well-known representative of such modal plants. At the same time, disease resistance is a key characteristic for cultivated plants; this process also appears to have a robust dynamics of growth of interest in the scientific literature (Fig. 10).

Thus, we conducted an analysis of the occurrence of genes of different organisms in the context of the disease-resistance process in the abstracts of PubMed, in which one of the three plants was presented: *Solanum tuberosum*, *Arabidopsis thaliana*, or *Zea ramose*. In particular, for *Solanum tuberosum*, we used the following query: a fixed organism (*Solanum tuberosum*), a fixed process (disease resistance), and any other organism or any gene. The keywords were not specified.

As a result of this query, in which *Solanum tuberosum* was a fixed organism, 21 abstracts were returned containing 32 genes and 43 organisms. In turn, for *Zea ramosa*, 19 abstracts were obtained with the mention of 35 genes and 43 organisms. A query for *Arabidopsis thaliana* revealed 143 abstracts with 231 genes and 86 organisms in total. Across all three cases, the ‘Filter by Nodes Score’ slider value was set to 5.

It appeared that among all detected organisms - 13 were presented across all three lists. Among them were phytopathogenic bacterium (*Pseudomonas syringae*), fungus-like eukaryotic microorganism (*Phytophthora infestans*), plant pathogenic fungus (*Rhizoctonia solani*), and gram-negative soil bacterium (*Agrobacterium tumefaciens*). The remaining eight organisms (rice; cotton; *Arabidopsis thaliana*; tomato; Tobacco; potato; wheat; maize; Barley) were plants of agricultural importance.

It is known that *Rhizoctonia solani* is a soil-borne fungal pathogen that is pathogenic to different host species [91, 92]. *Agrobacterium tumefaciens* is a ubiquitous representative of the soil microflora and can cause crown gall disease [93]. In turn, *Pseudomonas syringae* causes brown mucus, frostbite, fruit damage, and leaf spotting in plants [94]. *Phytophthora infestans* are phytopathogens that are known for causing late bligh [95].

**Fig. 11:**
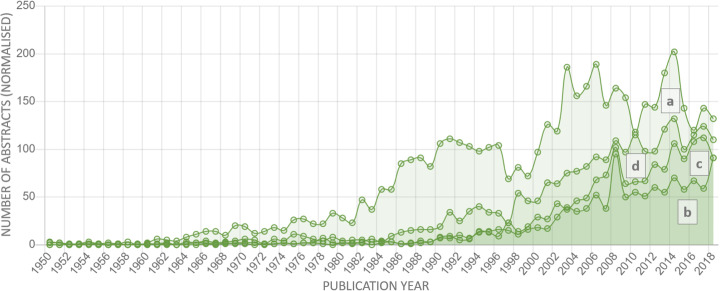
A normalized dynamics of growth of researcher’s interest in *Agrobacterium tumefaciens* (**a**) (trend strength 1310, p-value = 1.2E-11), *Phytophthora infestans* (**b**) (trend strength 1343, p-value = 3.34E-12), *Rhizoctonia solani* (**c**) (strength trend 1542, p-value = 3.33E-15), and *Pseudomonas syringae* (**d**) (trend strength 1747, p-value = 6.9E-20) by years

From Fig. 11, it can be seen that *Pseudomonas syringae* has the highest value for trend strength, which can indicate a growing interest in it from the research community. At the same time, the soil bacterium, *Agrobacterium tumefaciens*, has the highest number of references in the literature while the strength of this trend has the smallest value among all distributions presented. From the curve, it can be suggested that the highest number of mentions for this bacterium was manifested in the period from the 1990s to the beginning of the 2000s, after which it started to decrease. At the same time, during this period, there began an active growth of interest to three other represented organisms, which continues until now. Based on this graphical representation, we can conclude the importance of the tasks associated with fighting against diseases caused by the identified pathogens.

Among the found genes associated with resistance to diseases caused by the mentioned pathogens, snakin-1, Sgt1, and osmotin as examples can be distinguished. In particular, it is known that overexpression of the snakin-1 gene enhances the resistance of potato plants to *Rhizoctonia solani* [96]. The Sgt1 gene is a representative of single dominant resistance genes; the study shows that this gene plays a significant role in potato resistance to late blight disease [97]. Transgenic potato plants expressing wild osmotin proteins are known to become resistant to diseases caused by *Phytophthora infestans* [98].

In general, an analysis of the resulting list of genes showed that most of them are associated with the production of transgenic plants resistant to various diseases.

## Conclusions

The present work describes a new web-based ANDDigest module integrated into the ANDSystem tool, designed to search for information in pre-processed texts of PubMed abstracts with mapped objects from the ontology of the ANDSystem tool. The ontology of the ANDSystem tool features dictionaries for 13 types of objects, including molecular-genetics entities (genes, proteins, metabolites, microRNAs), cells and organisms, biological processes, diseases, drugs and their side effects, etc. Along with the objects represented in the ANDSystem tool dictionaries, the system provides the user with the ability to use their own keywords for the specification of search queries. ANDDigest is intended to facilitate the solution of two types of information search tasks: problems related to synonymy as well as the formation of search queries for finding documents that contain any names of objects of the type indicated in the request (from the corresponding dictionary) without the manual specification of their names. Search results can be presented in different forms: in the form of a table containing a mapped text or a network of interacting objects corresponding to the user request. Besides this, the GUI of ANDDigest allows filtering of search results according to various criteria, as well as sorting for abstracts and identified objects. A feature of the developed tool is providing the user with the ability to build graphical representations that describe the dynamics of interest for the recognized objects calculated on the basis of the frequencies of mentioning of the names of objects in scientific publications per year. The system also assesses the strength and statistical significance of trends assessed on the basis of these frequencies.

### Availability and requirements

**Project name:** The ANDDigest tool.

**Project home page:** https://anddigest.sysbio.ru/

**Operating system(s):** Platform independent.

**Programming language:** R, Perl, PHP v5.4, MySQL v5.7.25, JavaScript.

**Other requirements:** Internet connection, web-browser with HTML5 and CSS3 support.

**Any restrictions to use by non-academics:** None.

## Data Availability

The data sets supporting the results of this article are included within the article.

## Abbreviations

ANDSystem: Associative Network Discovery System
NER: Named-entity recognition
RE: Relationship extraction
SVM: Support vector machine
CRF: Conditional random field
HMM: hidden Markov model
KNN: K-nearest neighbor
BiLSTM: Bidirectional long short-term memory network
RNN: Recurrent neural networks
NLP: Natural language processing
POS: Part-of-speech
ML: Machine-learning
ANN: Artificial Neural Networks
BOW: Bag-of-words
GUI: Graphical user interface
ROC: Receiver operating characteristic
AUC: Area under receiver operating characteristic curve
SJR: Scientific Journal Rankings
XML: Extensible markup language
SVG: Scalable Vector Graphics
JSON: JavaScript Object Notation
CSV: Comma-Separated Values
PDF: Portable Document Format
PGF: Placental growth factor
